# The relationship between non-HDL cholesterol and macrophage phenotypes in human adipose tissue

**DOI:** 10.1194/jlr.P068015

**Published:** 2016-10

**Authors:** Rudolf Poledne, Ivana Kralova Lesna, Anna Kralova, Jiri Fronek, Sona Cejkova

**Affiliations:** Laboratory for Atherosclerosis Research, Centre for Experimental Medicine,* Institute for Clinical and Experimental Medicine, Prague, Czech Republic; Transplant Surgery Department,† Institute for Clinical and Experimental Medicine, Prague, Czech Republic

**Keywords:** atherosclerosis, inflammation, macrophage/monocyte, membrane receptors, high density lipoprotein

## Abstract

Data from experimental animal models and in vitro studies suggest that both hyperlipoproteinemia and obesity predispose to development of proinflammatory pathways of macrophages within adipose tissue. The aim of this study was to analyze whether non-HDL cholesterol concentration in healthy living kidney donors (LKDs) is related to the number and phenotype of proinflammatory macrophages in visceral and subcutaneous adipose tissue. Adipose tissue samples were collected by cleansing the kidney grafts of LKDs obtained peroperatively. The stromal vascular fractions of these tissues were analyzed by flow cytometry. Proinflammatory macrophages were defined as CD14^+^ cells coexpressing CD16^+^ and high-expression CD36 as well (CD14^+^CD16^+^CD36^+++^), while CD16 negativity and CD163 positivity identified alternatively stimulated, anti-inflammatory macrophages. Non-HDL cholesterol concentration positively correlated to proinflammatory macrophages within visceral adipose tissue, with increased strength with more precise phenotype determination. On the contrary, the proportion of alternatively stimulated macrophages correlated negatively with non-HDL cholesterol. The present study suggests a relationship of non-HDL cholesterol concentration to the number and phenotype proportion of macrophages in visceral adipose tissue of healthy humans.

The effect of cholesterol molecules on coronary heart disease (CHD) has been studied for more than a century with a substantial increase of the number of these studies coming after World War II (1). A high plasma LDL particle concentration is still considered one of the main risks of atherosclerosis (2). In the past two decades of the 20th century, molecular biology has documented that monocytes play a central role in this process. Also, epidemiological data have documented a certain risk of inflammation in CHD (3). At the same time, obesity and inflammation processes in adipose tissue have been shown to be major contributors to insulin resistance, diabetes, and CHD (4, 5). Recently, the relationship between monocyte behavior and the effects of cholesterol molecules was found to be bidirectional (6). It is now clear that not only do high atherogenic lipoprotein particles influence macrophage changes both in adipose tissue and the arterial wall (7), but also that the acute phase response downregulates reverse cholesterol transport (8).

Several direct effects of dietary cholesterol on local inflammation of adipose tissue have been described (6, 9) and reviewed (10) in experimental models. LDL particles, particularly mildly oxidized LDL, have been reported as being able to influence adipocyte differentiation, associated endoplasmic reticulum stress, and elevated mRNA of protein mediators of development of subclinical inflammation (11). In an experimental model using LDL receptor KO mice, alimentary cholesterol not only accelerated atherogenesis but also increased adipocyte hypertrophy, plasma concentration of the proinflammatory cytokine TNF-α and macrophage infiltration of adipose tissue (12). In an animal model sharing a very similar lipoprotein profile with humans, the pig, a hypercholesterolemic diet increased atherogenic lipoprotein and proinflammatory cytokine plasma concentrations and macrophage infiltration of visceral adipose tissue (12, 13). Based on these in vitro data and in vivo experimental models, it can be concluded that lipid overload influences adipose tissue development and proinflammatory functionality.

Much less is known about the response of adipose tissue to atherogenic stimuli in humans. Although adipose tissue has been shown to play an important role in insulin resistance and diabetes in humans (14, 15), macrophage status in adipose tissue has been described in detail in an animal model (16) but is still poorly understood in humans. High lipoprotein atherogenic risk in humans stimulates circulating monocyte adhesion (17, 18), their associated microparticle release (17), and a high subset of positively stimulated monocytes in the circulation (18, 19). In our present experiments, we were able to obtain macrophage phenotypes of adipose tissue from healthy human individuals and relate them to non-HDL cholesterol concentrations.

## METHODS

### Living kidney donors

All 47 individuals (enrolled between June 2013 and June 2015) were fully informed about the process of kidney donation and transplantation and, also, about adipose tissue sampling during organ cleaning before transplantation. All individuals signed informed consent forms and were interviewed with regard to their medical history and major cardiovascular risk factors. The design of the study was approved by the Ethics Committee of the Institute for Clinical and Experimental Medicine and Thomayer Hospital, Prague, Czech Republic.

### Tissue samples

**Fig. 1.**
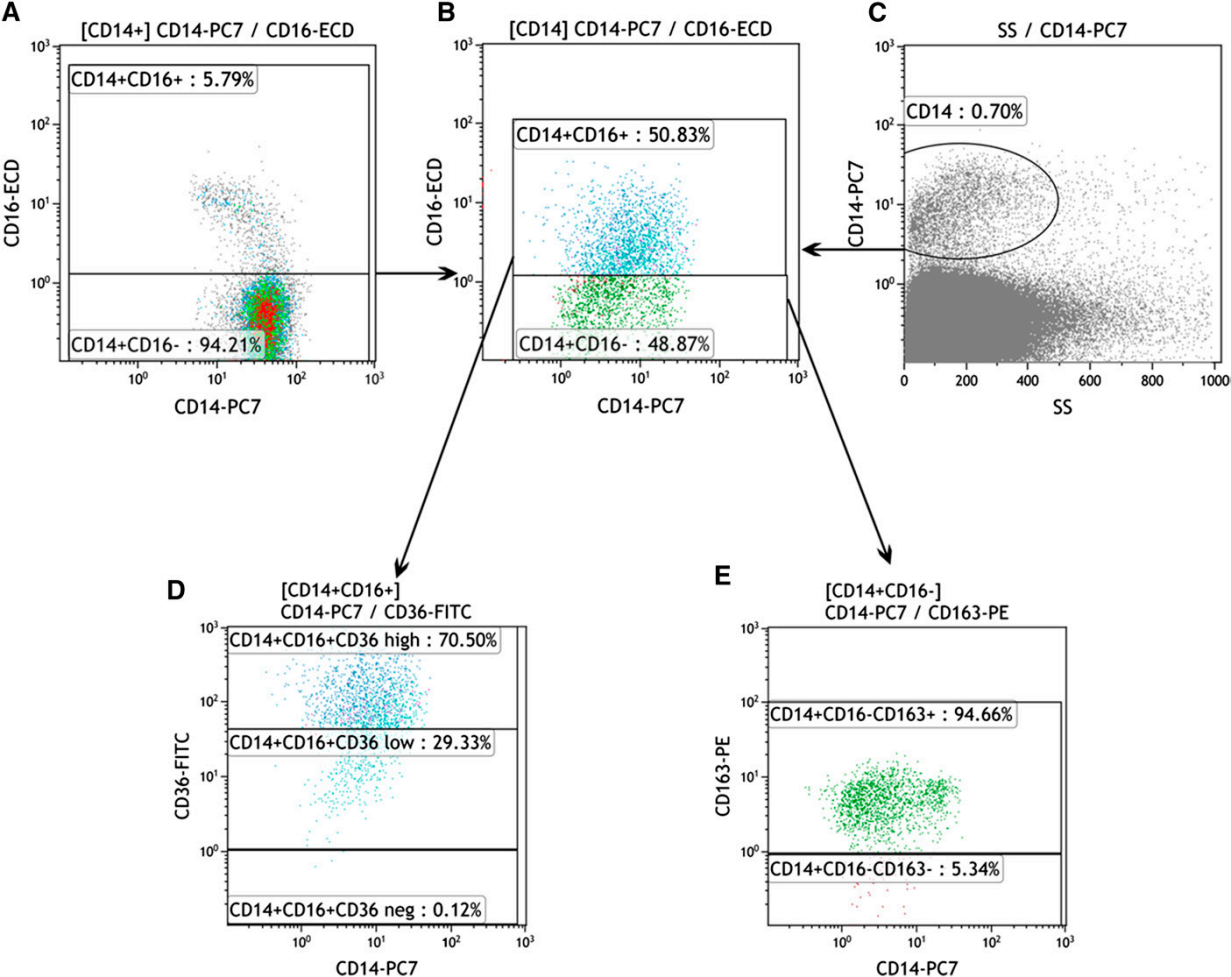
Example of SVF flow cytometric analysis. A: CD16-positive monocytes were first identified and delineated in the blood sample (left, CD16-positive macrophages in the upper part). The settings were fixed and subsequently used for SVF analysis (B). Total macrophages in SVF were identified by positivity for CD14 (C), and, based on the CD16 marker, two subpopulations were distinguished (B, CD16-positive macrophages in the upper part). The CD16^+^ subpopulation was divided according to the CD36 marker (D), with the highly positive subpopulation at the top and the low positive in the middle (based on blood macrophage analyses). E: CD163 expression was determined within the CD16-negative subpopulation and divided (CD163-positive macrophages in the upper part). This scheme is partly simplified, as a few minor fractions (already measured) are not mentioned.

Samples of visceral and subcutaneous adipose tissue were obtained peroperatively after hand-assisted laparoscopic nephrectomy. Adipose tissue samples (∼2 g) were immediately cooled and transferred to the laboratory within 20 min. After removing visible blood vessels and connective tissue, each sample was dissected using scissors to facilitate homogeneous collection of small pieces (∼2 mm^2^). After shaking incubation of tissue samples with collagenase (2 mg) for 20 min (37°C), the homogenate was filtered (50 μm) and centrifuged. The stromal vascular fraction (SVF) was purified twice by resuspension. The final SVF sample was analyzed immediately by flow cytometry (CyAn; Beckman Coulter, Brea, CA). Monoclonal antibodies and fluorochromes (CD14, Phycoerythrin-Cyanine, CD16, Phycoerythrin-Texas Red X, CD36, FITC and CD163 Phycoerythrin, PE/Clone RM 3/1) were used to define different subsets of monocytes/macrophages. Flow cytometry data were analyzed using Kaluza Software (Beckman Coulter). The viability of analyzed cells was measured for each sample using 7-AAD (7-Aminoactinomycin D), and only samples with a viability higher than 75% were considered. Due to difficulties in delineating CD16-positive cells in the SVF, CD16-positive monocytes were first identified and delineated in the blood samples where a CD16-positive subpopulation was clearly visible. The setting was fixed and subsequently used for SVF analysis. The gating strategy for identifying SVF macrophage subpopulations is shown in **Fig. 1**.

Based on data from the literature (19–21) and our recent results (22), we suggest that macrophages with a combined phenotype characterized by the expression of CD14 and CD16 and high expression of the phagocytic receptor CD36 (23) should correspond with normally stimulated M1 macrophages. On the other hand, macrophages with no CD16 expression, but with CD163 (24) positivity, might be considered anti-inflammatory M2 macrophages. At the same time, we are well aware that this classification could oversimplify the in vivo situation where the full phenotypic spectrum of transient phenotypes between M1 and M2 might exist. All other minority fractions represent <20% of the total of macrophages. The proportion of phenotype subpopulations was expressed as a percentage of all monocytes/macrophages. The absolute number of monocyte/macrophage cell lines per gram of adipose tissue was obtained by calculation of the dilution and amount of adipose tissue applied.

### Lipoprotein concentrations

Blood of kidney donors was obtained by venipuncture after 10 h fasting, immediately before surgery (prior to anesthesia induction). Samples were spun down within 30 min, and plasma was separated. Cholesterol and triglycerides were determined using a Hoffmann-LaRoche (Switzerland) enzymatic kit on a COBAS MIRA+ autoanalyzer. Concentrations of HDL fractions were analyzed using the same method after precipitation of apoB-containing particles with phosphotungstate. Non-HDL cholesterol concentrations were calculated as total cholesterol minus the HDL cholesterol fraction. Data of donors and population sample were analyzed using identical methods at the same laboratory (controlled quarterly by the Centers for Disease Control, Atlanta, GA).

### Statistical methods

Data are presented as means with SDs for continuous variables, and percentages with SDs for categorical variables. Intergroup comparisons of continuous variables and multiple linear regression adjustment were performed by the unpaired *t*-test with JPM 10.0 software. Correlation analysis was performed using biostatistical GraphPad Prism software, version 6 (GraphPad Software Inc., San Diego, CA). In all tests, *P* values higher than 0.05 were considered statistically nonsignificant.

## RESULTS

The study group of living kidney donors (LKDs) included individuals who were slightly healthier compared with a well-selected representative population sample, WHO MONICA Study (25). The BMI of the LKD group was slightly yet significantly lower compared with the representative population sample (**Table 1**). Total cholesterol concentration and non-HDL cholesterol concentration were much lower in LKDs (13% and 14%, respectively). Prevalence of hypercholesterolemia (>5 mM) and high non-HDL cholesterol concentration was much lower in LKDs (13% and 14%, respectively). Five LKD individuals had dyslipoproteinemia, and five individuals were treated with statin and two received antihypertensive drugs.

**TABLE 1.** Characteristics of LKDs group (men, n = 17; women, n = 30)

Characteristics	N	Mean ± SD
Age (years)	47	46.05 ± 10.60
BMI (kg m^−2^)	47	25.75 ± 3.58
Cholesterol (mM)	47	4.40 ± 0.95
HDL cholesterol (mM)	47	1.16 ± 0.38
Non-HDL cholesterol (mM)	47	3.24 ± 0.92
Triglycerides (mM)	47	1.45 ± 0.80

Data are expressed as mean ± SD.

**Fig. 2.**
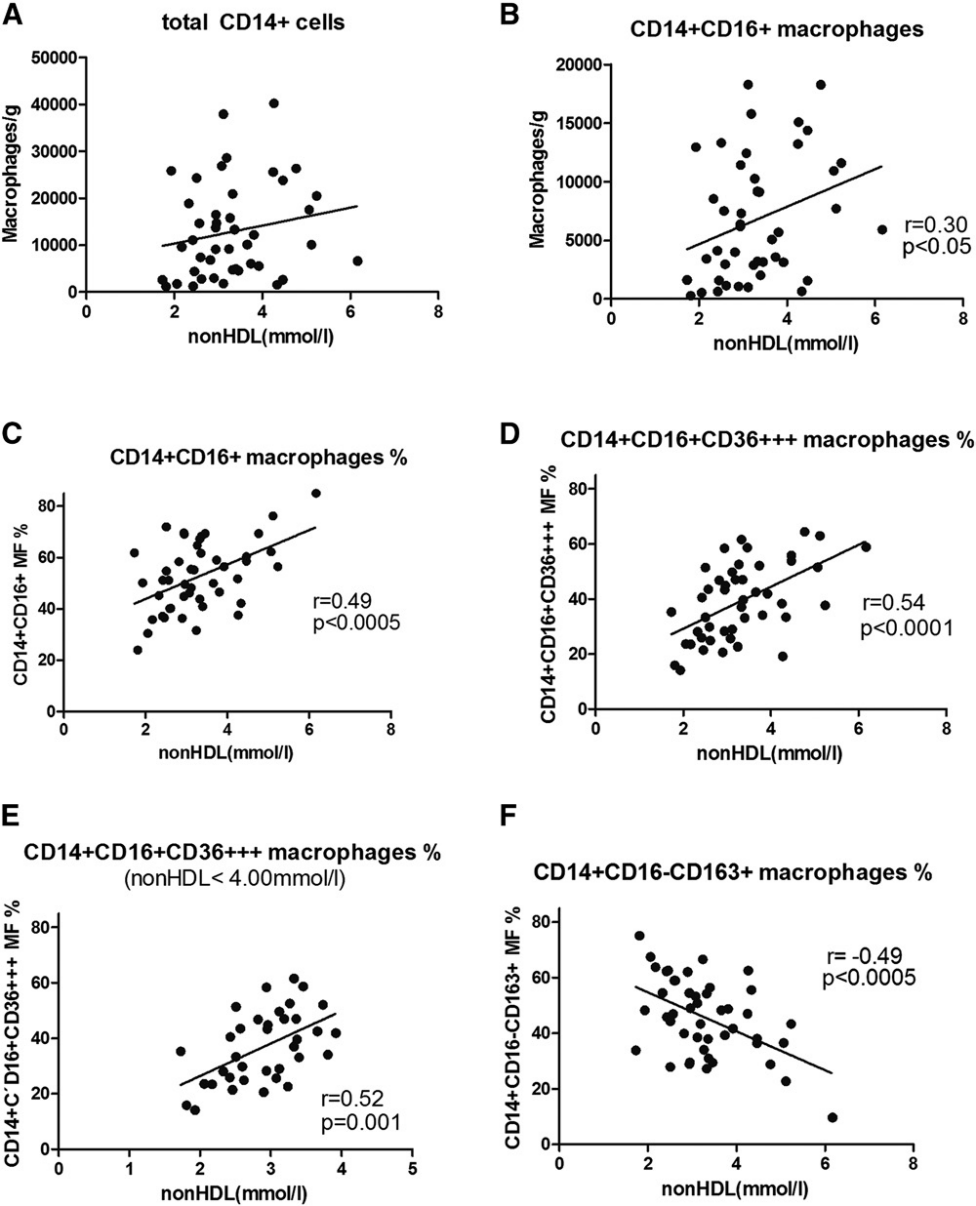
The relations between different macrophage subpopulations and non-HDL cholesterol concentration in visceral adipose tissue. Correlations of macrophage number and proportion of phenotypes with non-HDL cholesterol in the visceral adipose tissue of 47 LKDs (or their subset in E). Consequently, total CD14^+^ cells (A), CD14^+^CD16^+^ cells (B), CD14^+^CD16^+^ % (C), CD14^+^CD16^+^CD36^+++^ % (D), CD14^+^CD16^+^CD36^+++^ % (limitation 2–4 mM) (E), and CD14^+^CD16^−^CD163^+^ % (F).

The total number of macrophages per gram of visceral adipose tissue was not significantly related to non-HDL cholesterol in 47 LKDs (**Fig. 2A**). A borderline level of significance was observed when correlating normally polarized proinflammatory CD16^+^ macrophage number to non-HDL cholesterol (Fig. 2B). This level of significance substantially increased when expressing the proportion of these macrophages as the percentage of all macrophages in the sample (*P* < 0.005; Fig. 2C). When including an additional surface marker of the normally polarized macrophages, high CD36 (CD36^+++^) expression, in the correlation analysis, the correlation of macrophages to non-HDL cholesterol became very close (*P* < 0.001; Fig. 2D). To determine whether this correlation was expressly produced by the five individuals with the highest concentrations of non-HDL cholesterol, those with levels >4 mM were excluded from the subsequent calculation (Fig. 2E). Although this correlation was limited to individuals within a very narrow range of non-HDL cholesterol between 2 and 4 mM, the significance of the correlation of the proportion of CD16^+^, CD36^+++^ to non-HDL cholesterol remained unchanged (*P* < 0.001). Finally, after calculating the correlation of the proportion of alternatively polarized macrophages to non-HDL cholesterol concentrations, a significant inverse correlation was found (Fig. 2F). All these relationships were also analyzed when the number of macrophages and their proportions were adjusted for sex, age, and BMI. This analysis by multiple linear regression method (JMP 10.0) did not document any effect of these parameters, and the relationship of all macrophage phenotypes to non-HDL cholesterol concentration remained at the same level of significance with the exception of a negative correlation of alternatively stimulated macrophages (CD14^+^CD16^+^CD163−; Fig. 2F), where the level of significance was only 0.002 compared with original the 0.0005.

**Fig. 3.**
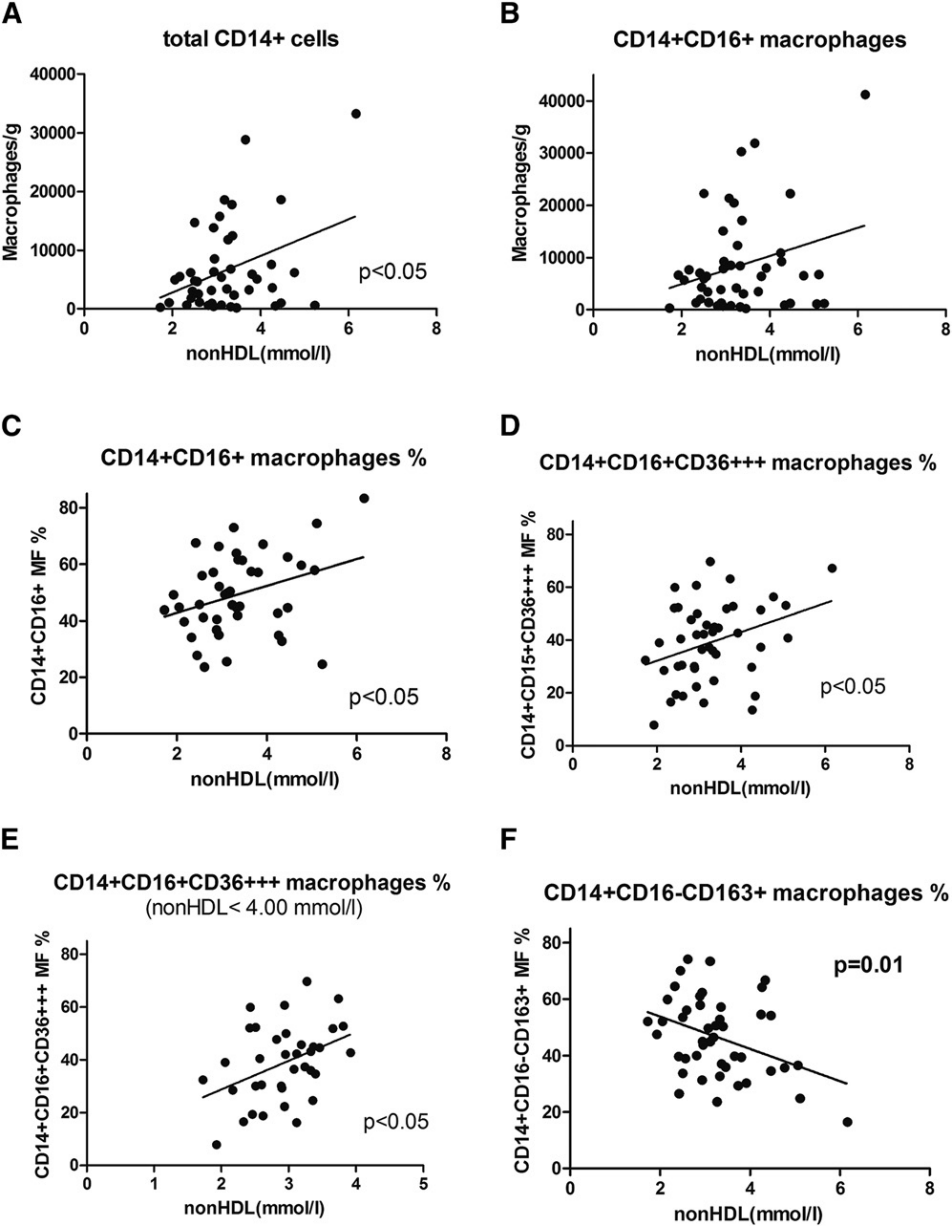
The relations between different macrophage subpopulations and non-HDL cholesterol concentration in subcutaneous adipose tissue. Correlations of macrophage number and proportion of phenotypes with non-HDL cholesterol in the subcutaneous adipose tissue of 47 LKDs (or their subset in E). Consequently, total CD14^+^ cells (A), CD14^+^CD16^+^ cells (B), CD14^+^CD16^+^ % (C), CD14^+^CD16^+^CD36^+++^ % (D), CD14^+^CD16^+^CD36^+++^ % (limitation 2–4 mM) (E), and CD14^+^CD16^−^CD163^+^ % (F).

Although the correlation pattern of the total macrophage and CD14^+^CD16^+^ macrophage contents (**Fig. 3A, B**) and the proportion of CD14^+^CD16^+^ and normally stimulated macrophages CD14^+^, CD16^+^, CD36^+++^ (Fig. 3C, D) of subcutaneous adipose tissue to non-HDL cholesterol were similar to those of visceral tissue, the significance was much lower (only *P* < 0.05) and only borderline in all relations. Also, the correlation of alternatively stimulated macrophages in subcutaneous adipose tissue to non-HDL cholesterol exhibited the same pattern as visceral adipose tissue, but with a lower level of significance (Fig. 3F).

## DISCUSSION

Inspired by a review by Aguilar and Fernandez (11) summarizing recent data on the cholesterol/macrophage relationship both in obesity and nonobesity experimental models, we sought to analyze the relationship between proinflammatory macrophage status of adipose tissue and cholesterolemia in healthy men and women. Analysis of this type of proinflammatory status by determining macrophage phenotypes in adipose tissue is rather complicated as there is no definitive identification of surface markers for normally and alternatively polarized macrophages. In recent literature, the presence of the CD16 receptor together with CD14 has been considered for identifying proinflammatory, normally polarized macrophages (26). On the other hand, the CD16^+^ marker on the surface of circulating monocytes has been suggested to belong to alternatively stimulated macrophages (18, 23). One can hardly accept the possibility of a substantial change of circulating monocytes from pro- to anti-inflammatory phenotypes upon invading adipose tissue and their eventual transformation to macrophages. Based also on our earlier data (22), we suggested CD16 positivity as a marker of the proinflammatory phenotype. To develop a more precise definition of the proinflammatory phenotype, we analyzed the presence and absence of the CD36 receptor. We found a more conclusive relationship between non-HDL cholesterol and normally polarized macrophages (CD14^+^CD16^+^) when high CD36 positivity (CD36^+++^) was included. When considering all CD14^+^CD16^+^ macrophages coexpressing also any CD36 marker, the relationship between normally polarized macrophages and non-HDL cholesterol almost disappeared. The CD36 receptor, also referred to as the scavenger receptor B2, plays an important role in the transfer of cholesterol molecules across the macrophage plasma membrane and an even more important role in reverse cholesterol transport (8). It clearly follows from this finding that this receptor is an important part of membrane function of all macrophages.

In this study, our data document that non-HDL cholesterol concentrations in subjects are positively related to the proportion of proinflammatory macrophages in visceral adipose tissue. The correlation coefficient increases with a more precise definition of proinflammatory phenotypes in the following order: CD14^+^, CD16^+^, and CD36^+++^. This relationship was not dependent on the individuals with the highest non-HDL cholesterol levels and remained close (*P* < 0.001) after excluding hypercholesterolemic LKD individuals.

Data on subcutaneous adipose tissue macrophages in humans were published for the first time only recently (27), and, surprisingly, an unexpected prevalence of anti-inflammatory monocyte concentrations was found in obese individuals compared with lean controls. It is possible that differences between subcutaneous and visceral adipose tissue in our study might be explained in this way. Although the cause of the correlation of non-HDL cholesterol concentration with proinflammatory macrophages in adipose tissue is not clear and therefore can be only speculative, these relations are expressed both in the subcutaneous and visceral adipose tissue. All types of these relations of non-HDL cholesterol concentration and different phenotypes of proinflammatory macrophages (as shown in Figs. 2 and 3) are, however, much less pronounced in subcutaneous compared with visceral adipose tissue. This is not consistent with the generally accepted unique role of visceral adipose tissue in metabolic regulation and in the adipose tissue-driven subclinical inflammation status.

The probable precursors of proinflammatory tissue macrophages, circulating monocytes with the same surface marker, were found to have a lower capacity for reverse cholesterol transport in a large group of patients with chronic kidney disease (18). In addition, the high number of CD14^+^, CD16^+^ circulating monocytes predicted cardiovascular risk in these patients (and has been conclusively shown to be the main cause of death). Not surprisingly, the documented number of “proinflammatory monocytes” was a much stronger predictor of atherosclerosis-related complications compared with HDL cholesterol or apoA1 concentrations. It has been documented that HDL cholesterol concentration does not correspond with the real rate of reverse cholesterol transport (28). The decrease of circulating proinflammatory monocytes has been proved after rosuvastatin treatment, a positive change potentiated by synergic exercise (26). Also, diet influences the proportion of monocytes considered proinflammatory along with the combination of a healthy diet pattern with a lower proportion of proinflammatory phenotypes (29). In addition, high plasma cholesterol contractions in patients with familial hypercholesterolemia were found to be associated with a high proportion of circulating CD14^+^ and CD16^+^ monocytes and monocyte-derived microparticles (17) compared with levels documented in a healthy control group. All these data regarding circulating monocytes with identical surface markers are in agreement with ours, and it is likely that these cells are precursors of proinflammatory macrophages in adipose tissue.

Our data of the strong relationship of plasma cholesterol concentration to inflammation in fact confirm the bidirectional effect of cholesterol molecules and proinflammatory macrophages as suggested by experimental models (6–8) and tissue culture studies. This could be a potential explanation of our results with substantial involvement of oxidized LDL seen in experimental models. However, there is a substantial difference between hyperlipoproteinemia induced by crystalline cholesterol supplementation to the diet used in experimental models (10, 12) and in vitro studies (11), compared with non-HDL cholesterol concentrations in humans, which fell within the normal range in the majority of LKD subjects. Also analyzed were the relationships of CD14^+^, CD16^+^, and CD36^+++^ macrophage proportions to total cholesterol concentration in visceral adipose tissue. Although a positive correlation was found, its level of significance was fairly low (*P* < 0.05). There was no relationship of macrophage phenotypes to the concentration of HDL cholesterol.

Although the reason for the high correlation of non-HDL cholesterol concentration to proinflammatory macrophages in the visceral adipose tissue has not been clearly identified, this fact might have high biological importance. In particular, increasing significance of this relationship with increasing description of proinflammatory phenotype of macrophages should be considered. Increasing proinflammatory status of adipose tissue of individuals with high non-HDL cholesterol concentrations may represent the synergic proatherosclerotic status of these individuals.

Individuals in the highest quintile of non-HDL cholesterol concentration of our study group have been shown to have twice as high a proportion of CD14^+^, CD16^+^, and CD36^+++^ macrophages compared with those in the lowest quintile. Similarly, the proportion of the anti-inflammatory macrophages CD14^+^, CD16^−^, and CD163 in the highest quintile of non-HDL cholesterol concentrations is only one-half of this macrophage phenotype compared with the lowest non-HDL cholesterol concentration quintile.

Independently of the mechanism, the close relationship of non-HDL cholesterol concentration to the proinflammation status of human visceral adipose tissue is consistent with the recently summarized data on cholesterol and inflammation in experimental models (8).
